# Microbiological, Health and Comfort Aspects of Indoor Air Quality in a Romanian Historical Wooden Church

**DOI:** 10.3390/ijerph18189908

**Published:** 2021-09-20

**Authors:** Florin Marcu, Nicolaie Hodor, Liliana Indrie, Paula Dejeu, Marin Ilieș, Adina Albu, Mircea Sandor, Cosmin Sicora, Monica Costea, Dorina Camelia Ilieș, Tudor Caciora, Anca Huniadi, Iuliana Chiș, Lucian Barbu-Tudoran, Paul Szabo-Alexi, Vasile Grama, Bahodirhon Safarov

**Affiliations:** 1 Faculty of Medicine and Pharmacy, 10 Piața 1 Decembrie Street, 410073 Oradea, Romania; Marcu.Florin@didactic.uoradea.ro (F.M.); drims75@yahoo.com (M.S.); ancahuniadi@gmail.com (A.H.); 2 Faculty of Geography, Babes-Bolyai University, 5-6 Clinicilor Street, 400006 Cluj Napoca, Romania; nicolaie.hodor@ubbcluj.ro; 3 Department of Textiles, Leather and Industrial Management, Faculty of Energy Engineering and Industrial Management, University of Oradea, 4 Barbu Stefanescu Delavrancea Street, 410058 Oradea, Romania; lindrie@uoradea.ro (L.I.); aalbu@uoradea.ro (A.A.); 4 Medical Laboratory Service M.D., Bethany Medical Clinic Oradea, Bihor County, 410004 Oradea, Romania; office@betania-centrumedical.ro; 5 Faculty of Geography Extension, Babes-Bolyai University, 6 Avram Iancu Street, 435500 Sighetu Marmatiei, Romania; marin.ilies@ubbcluj.ro; 6 Biological Research Center Jibou, 16 Wesselenyi Street, 455200 Jibou, Romania; cosmin.sicora@gmail.com (C.S.); simeoni_iulia@yahoo.com (I.C.); 7 Faculty of Environmental Protection, University of Oradea, Gen Magheru Street, 410048 Oradea, Romania; mcostea@uoradea.ro; 8 Department of Geography, Tourism and Territorial Planning, Faculty of Geography, Tourism and Sport, University of Oradea, 1 Universitatii Street, 410087 Oradea, Romania; dilies@uoradea.ro (D.C.I.); szaboalexipaul@gmail.com (P.S.-A.); vgrama@uoradea.ro (V.G.); 9 Faculty of Biology and Geology, Babes-Bolyai University of Cluj Napoca, 5-7 Clinicilor Street, 400006 Cluj-Napoca, Romania; lucian.barbu@itim-cj.ro; 10 National Institute for R&D of Isotopic and Molecular Technologies, 67-103 Donat Street, 400293 Cluj-Napoca, Romania; 11 Department of Digital Economy, Samarkand State University, Samarkand 140104, Uzbekistan; safarovb@rambler.ru

**Keywords:** indoor microclimate, temperature, humidity, carbon dioxide, fungi, heritage building, biodeterioration, public health

## Abstract

Monitoring the indoor microclimate in old buildings of cultural heritage and significance is a practice of great importance because of the importance of their identity for local communities and national consciousness. Most aged heritage buildings, especially those made of wood, develop an indoor microclimate conducive to the development of microorganisms. This study aims to analyze one wooden church dating back to the 1710s in Romania from the microclimatic perspective, i.e., temperature and relative humidity and the fungal load of the air and surfaces. One further aim was to determine if the internal microclimate of the monument is favorable for the health of parishioners and visitors, as well as for the integrity of the church itself. The research methodology involved monitoring of the microclimate for a period of nine weeks (November 2020–January 2021) and evaluating the fungal load in indoor air as well as on the surfaces. The results show a very high contamination of air and surfaces (>2000 CFU/m^3^). In terms of fungal contamination, *Aspergillus* spp. (two different species), *Alternaria* spp., *Cladosporium* spp., *Mucor* spp., *Penicillium* spp. (two different species) and *Trichopyton* spp. were the genera of fungi identified in the indoor wooden church air and *Aspergillus* spp., *Cladosporium* spp., *Penicillium* spp. (two different species) and *Botrytis* spp. on the surfaces (church walls and iconostasis). The results obtained reveal that the internal microclimate not only imposes a potential risk factor for the parishioners and visitors, but also for the preservation of the wooden church as a historical monument, which is facing a crisis of biodeterioration of its artwork.

## 1. Introduction

Indoor air quality (IAQ) monitoring has become a necessary practice due to the risks it poses for human health. Specialized studies focus on indoor microclimate as well as the load of pathogens or hazardous pollutants [1,2]. Older constructions and heritage attractions are known to have such high fungi loads particularly if insufficient attention is paid to the monitoring and control of the indoor microclimate around the three elements of heating-ventilation-lighting. Additional problems are caused by the construction material (especially wood), which forms a culture medium for the growth of microorganisms and fungi. Solid wood buildings can present microbial load by means of direct dust settlement due to a complex combination of local environmental conditions [3,4]. Arguably, old heritage buildings must be monitored in order to minimize the risks to the health of visitors and employees, to optimize the maintenance costs, and prior to their inclusion in sightseeing tours.

This paper undertakes a multi-layered analysis of air quality in a heritage wooden church, from the point of view of microclimate and fungal load. Three core questions are addressed: (1) whether there is a hazardous fungal load in comparison to acceptable levels; (2) what is the evolution pattern of indoor microclimate parameters in various weather conditions and seasons; (3) whether it is possible to identify areas and periods which are safe for sightseeing.

Studies related to IAQ show differences between the short-term effects of contact with various agents, the long-term effects, and unpleasant odors. The effects of short-term exposure and the effects generated by unpleasant odors are important for sightseeing [5,6]. Contact can be made by inhalation or by touching exposed surfaces. Therefore, this study combines data obtained from direct measurements of IAQ and data generated from laboratory tests performed on air and surface samples.

The wooden church of Boianu Mare (Bihor County, Romania) is the focus of investigation, an 18th century heritage building in good state of conservation. It is an attraction for cultural tourism tours because of its size and the nature of its conservation [7,8,9]. The church has been used for several years only on special occasions, for which little funds have been allocated for heating during the winter season, as well as for other necessary maintenance operations, and consequently fungal contamination has occurred [10].

**Figure 1:**
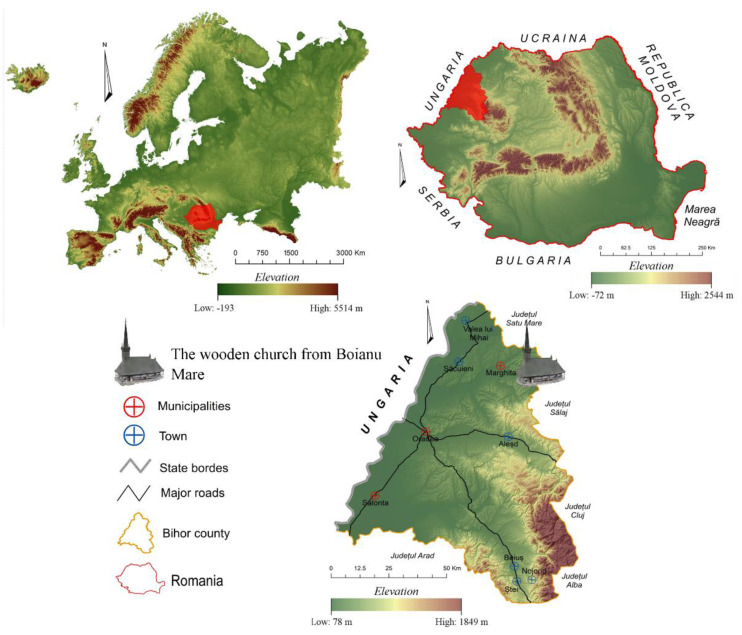
The location of the Wooden Church from Boianu Mare at the scales of Europe, Romania and Bihor County.

**Figure 2:**
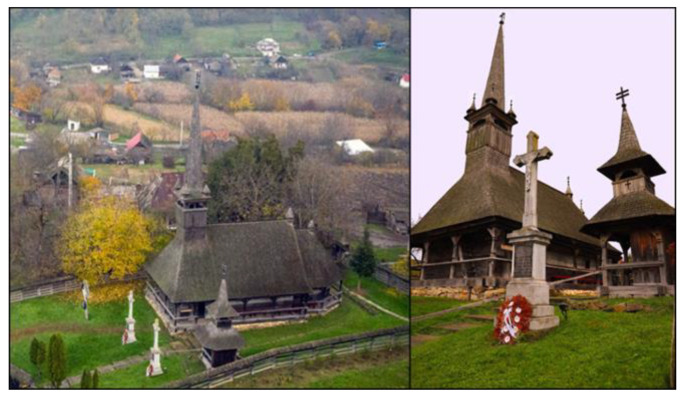
Overview of the exterior of the monument.

The wooden church is dedicated to Saint Archangels Michael and Gabriel and was classified as a historical monument in the year 2010. It functions as a Greek Catholic parish church, located in a central area in the village of Boianu Mare (Figure 1 and Figure 2). The building has a well-defined scale and is built in the Gothic style typical of wooden churches located in Bihor County. The foundation is made of a carved stone wall. The east-west oriented building was constructed in 1710 by using massive oak beams on the site of a previous church for which, inside, religious items dating back to 1686 were found and, on the beams, various signs of reassembling during building construction are evident [11,12,13].

## 2. Literature Review

Conservation of cultural heritage plays a major part in protecting heritage spaces for future generations. Critical conservation issues arise around the religious and spiritual values of monasteries and churches as well as valuable items kept inside these buildings (iconostasis, worship pots, icons, textiles, books, etc.). Among microorganisms, fungi are the main biological agents that cause the biodeterioration of religious artefacts in such churches and also represent a risk factor for human health.

The existing international literature contains several relevant studies. Pepe et al. [14] analyze the heterotrophic microorganisms found in medieval paintings in seven historic churches in Campania, Italy. Sterflinger et al. [15] provide an overview of biodeterioration phenomena on different materials and fabrics: stone and building materials, and objects exhibited or stored in the museums and libraries. Ortiz et al. [16] identify the genera of fungi that contribute to wood deterioration by studying eight wooden churches in Chile. Frankl [17] presents the results of a comparative study on the biological damage to small and medium-sized Baroque churches in the Czech Republic. Fungal contamination of fabric items preserved in Slovenian museums and churches was studied by Kavkler et al. [18]. Similar research was conducted by Kalamees et al. [19] and emphasized problems related to the indoor climate which causes the risk of damage in naturally ventilated churches in the cold climate of Estonia. Di Carlo et al. [20] investigated bacterial and fungal colonization in three closed environments with different structures and different temperature and humidity conditions: archive, cave and hypogeum. Their research highlights the microorganisms capable of inducing the deterioration of artefacts, as well as being potentially dangerous to human health. Chmiel et al. [21] investigate bacterial and fungal aerosols in ten historical attractions from Kraków (churches, crypts, museums and libraries). Research conducted by Nawalany et al. [22], Stanaszek-Tomal [23] and Mang et al. [24], addresses issues related to hygrothermal conditions in churches, with emphasis on air temperature and humidity which periodically exceed the values of thermal comfort for historical buildings and exert a negative impact. Microorganisms present on religious items are responsible for these physical and chemical changes.

For thousands of years, fungi have played an important role in relation to human culture and the evolution of our society. In contrast to the numerous beneficial effects, fungi also have many disadvantages including their role as allergens, mycotoxins, in food spoiling, pathogenicity and biodeterioration of materials [25], and their negative effects for cultural heritage are noteworthy because of their enormous enzymatic activity. In recent decades, a challenge has emerged for restorers, architects and researchers on the development of appropriate treatments for contaminated objects and the prevention of fungal growth. Fungi can inhabit, alter and degrade all types of organic and inorganic materials [26]. The inactivation of fungal and bacterial species by ultra-violet radiation (UV) has been known for a long time [27]. The tested fungicidal effect of UV on different yeast strains showed that UV sensitivity is dependent on the fungal strain [28].

In Romania, there are several studies that aim to determine the bacteriological microflora and the internal microclimate in wooden churches, as well as historical monuments, both in order to preserve them and limit negative effects on human health. Lupan et al. [29] investigated the fungal load in the wooden church dating back to the 17th century in Nicula Monastery (Cluj County). Gomoiu et al. [30] focus on a wooden church from Amărăști, Vâlcea County. Oneț et al. [31] and Ilieș et al. [32,33] determined the microbiological contamination in relation to the microclimate conditions in the wooden church *“St. Martyrs Constantin Brancoveanu and His Sons”* from the campus I of the University of Oradea. Microbiological contamination and its implications on cultural heritage frescoes were studied by Radu et al. [34], Mohanu et al. [35], Bucsa and Bucsa [36] and Ilieș et al. [37]. Further, other relevant studies focus on the determination of fungal contamination and microclimatic problems in museums [13,38,39,40,41], sport halls [42,43] or schools [44,45,46]. These studies have shown that among the most common fungi that can be identified in the old wooden churches on the Romanian territory are *Aspergillus* spp., *Mucor* spp., *Arthrinium* spp., *Stachybotrys* spp., *Scopulariopsis* spp., *Penicillium* spp., *Absidia* spp., *Geotrichum* spp. or *Rhizopus* spp.

## 3. Materials and Methods

The research methodology involved collecting samples from air and surfaces inside the wooden church, as well as monitoring of the indoor microclimate. The purpose of these procedures was to determine favorable or unfavorable conditions for human activity within the historical monument and for the preservation of the monument.

**Figure 3:**
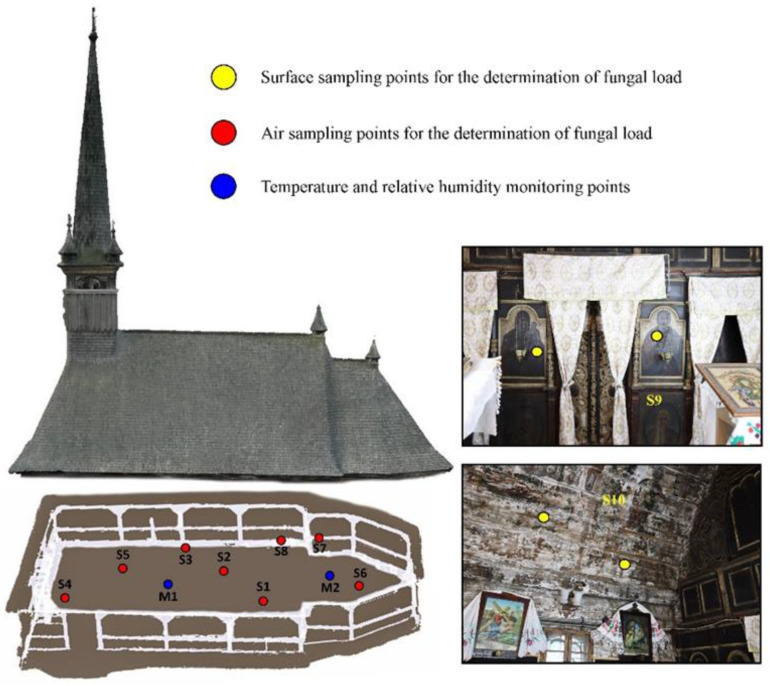
Spatial distribution of sampling points for the assessment of the fungal load and the location of the devices for determining the indoor microclimate.

The main indicators of the indoor microclimate, i.e., temperature and relative humidity (RH), were monitored for a period of nine weeks, between November 2020 and January 2021. For the monitoring activity, Klimalogg Pro data-logger sensors were used, set for the automatic measurement of values every hour. The thermo-hygrometer sensors record and store data with an accuracy of ±0.1 °C for temperature and ±3% for RH. Due to the relatively small differences between the rooms inside the wooden church, it was decided to place one sensor in the nave and another in the altar (Figure 3).

For this study, the winter period was chosen because it is problematic, considering that the church does not have a heating, ventilation and air conditioning system (HVAC); this means that the indoor microclimate is mostly conditioned by the outside weather. At the same time, in this season the biggest variations of the microclimatic parameters (very high humidity associated with low temperatures) can occur, which can cause degradation of objects over time and the appearance of bacteriological microflora. All this, combined with the fact that the winter season is the peak time of the year in terms of liturgical services organized indoors (due to the winter holidays), creates unfavorable conditions for human health. These conditions can lead, against the background of low immunity during this period [47,48], to the easier contacting of different pathogens, both due to the microclimatic conditions and due to the bacteriological microflora present inside.

In order to secure more accurate results regarding the microbiological contamination of the air, eight sampling points were applied with Petri dishes seeded with sterile Sabouraud culture medium. The Petri dishes were evenly distributed so that samples could be taken from the narthex, the nave and the altar (Figure 3). These were exposed for 30 min, at a height ranging between 1.5 and 1.7 m, according to the average human height. The assessment of the airborne fungal load inside the historical wooden church was performed by the Koch sedimentation method [49], which is based on the passive sedimentation of airborne particles due to gravity.

Samples from the surface of the examined items were collected using sterile cotton swabs [50,51], the sampling surface being approximately 1 cm^2^ for each of the samples. Cotton swabs were treated by immersion in one milliliter of sterile water. Thereby, four samples were collected from the surfaces of the paintings placed on the iconostasis and on the wall of the nave. The material from which the samples were taken was both cotton canvas and wood. Each sample taken was then inoculated into a nutrient agar on Petri dishes to identify bacteria, and also in Sabouraud agar containing chloramphenicol to isolate fungal genera [52]. After incubation at a temperature of 28 °C (in the incubator), the final identification of the fungi was made through examining of the macroscopic and microscopic characteristics of the grown colonies [53,54]. They became macroscopically visible after 24 h; afterwards, they entered a maturation process, with changes in diameter, shape, color and texture, some genera completely invading the culture medium. Quantitative analysis of the degree of fungal contamination of air was performed by counting the colonies which had grown on the culture medium, expressed in CFU/mL, and equating the result in terms of air volume using two formulae, namely the Polish standard PN 89/Z-04008/08 and Omelianski’s formula [55,56].

Omelianski’s formula is based on the observation whereby on a surface of 100 cm^2^ exposed to air for a certain period of time a number of microorganisms equal to that contained in a volume of 10 dm^3^ air achieves sedimentation.

CFU/m^3^ air = n × 10,000/S × k, where n is the number of colonies grown on the surface of the dish, S is the surface of the Petri dish (for a 9 cm diameter, S = 3.14 × R^2^ = 63.5 cm^2^) and k is the coefficient of exposure time to air (expressed in minutes, k = 1 for 5 min; k = 2 for 10 min; k = 3 for 15 min, etc.).

The Polish Standard PN 89/Z-04008/08 Formula is

CFU/m^3^ air = n × 10,000/S × t × 0.2, where n is the number of Petri dishes used, S is the surface of the Petri dish in cm^2^ (for a 9 cm diameter, S = 3.14 × R^2^ = 63.5 cm^2^) and t is the exposure time of the dish.

For cleaning the inner surfaces to remove harmful microbiological flora, a good and non-invasive solution is represented by treatment with ultraviolet radiation of type B (UVB) (with wavelengths between 290 and 320 nm). For testing this solution, different canvas samples (from hemp) were collected from the wooden church in Boianu Mare, considering that, according to Sterflinger [15], among the most frequent hyphomycetes in museums and on materials of objects of arts are fungal strains, such as *Alternaria* spp., *Aspergillus* spp., *Fusarium* spp. and *Cladosporium cladosporioides*. For an easier application, two different species of fungi were chosen (*Aspergillus tamarii* and *Cladosporium cladosporiodes*) to be artificially inoculated and carefully grown on the pieces of canvas collected from inside the monument. Each had a suspension of fungal strain in 1 mL of distilled water. In total 20 µL of suspension was added to each Petri plate.

In order to test the UVB radiations effect on the fungal strains, after an incubation period of 5 days, two samples were irradiated for 30 min each at a maximum power of 315 nm, while two other seeded samples were kept as a reference. The experiments were carried out in a laboratory room controlled in terms of temperature and relative humidity (25 ± 1 °C, respectively between 40–50% RH), using two 15W ROTH IV type UVB lamps, with a wavelength between 254 and 366 nm. Before and after the completion of the irradiation of the materials, they were analyzed by Scanning Electron Microscopes (SEM) to identify any changes in the surface and the fungal load. The device used for this purpose was FEI Quanta 200 microscope, the images being rendered at a resolution of 30.0 kV.

## 4. Results

For the proper preservation of indoor items and the comfort of parishioners, the parameters of the indoor microclimate should not vary greatly and ideally should be kept within a limited range [31]. In compliance with American Society of Heating, Refrigerating and Air-Conditioning Engineers (ASHRAE) standards [57], the indoor microclimate is ideally maintained at an average value of 20 °C (±1–2 °C) in terms of temperature and 50% (±3%) in the case of relative humidity (RH).

**Figure 4:**
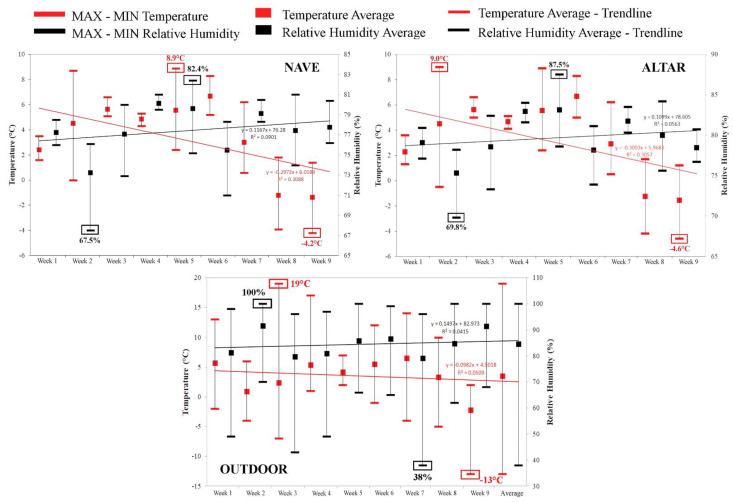
Fluctuations in the minimum, maximum, and average weekly temperature and RH values within the nave and altar of the historic wooden church in Boianu Mare, Romania.

The indoor microclimate is entirely dependent on the outdoor conditions, since the church does not have HVAC systems. Thus, the average temperature in the nave during the nine weeks of monitoring was 3.34 °C, while in the altar temperature registered 3.27 °C. The average values of relative humidity throughout the entire analysed period are 77.45% in the nave and 79.70% in the altar (Figure 4). These values do not meet the above mentioned standards and importantly represent a potential hazard to the health of parishioners and to the integrity of the items found inside the church.

The parameters of the indoor microclimate are closely correlated with that of the outside. The data taken from the nearest weather station (Sacuieni Weather Station, 33 km away from the monument), reveals that (as inside) the outdoor humidity is on a slight upward trend, while the temperature gradually decreases. In the case of external humidity, the maximum value (100% RH) was met several times in the studied interval, including in week 5, when the maximum value was recorded inside. In terms of temperature, weeks 3–5 coincided with the highest temperatures both indoors and outdoors; and the last week was the coldest with negative average values in both situations (Figure 4).

The combined effect of temperature and RH make the indoor microclimate unsuitable for human activity and the conservation of items located inside the church (such as paintings on wood and canvas, icons and other artefacts). At the same time, the extremely high humidity values favour the development and multiplication of fungi and bacteria colonies [58].

All fungal colonies grown on culture media were part of the category of molds. There are currently no universally accepted standards or regulations regarding the airborne fungal load; therefore, for the assessment of the degree of air contamination inside rooms, certain indicative rules were established as shown on Table 1 [59].

The calculation method involves the arithmetic mean of the colonies on the Sabouraud medium corresponding to the examined room, the interpolation in Omelianski’s formula, respectively the formula according to the PN 89/Z-04008/08 standard, and interpretation of the results within one of the five degrees of contamination according to Table 1. An example of calculation could be achieved using dish 7, on which the lowest number of different colonies has grown within 48 h, respectively 82 h.

Omeliansk’s formula: CFU/m^3^ air = 82 × 10,000/63.5 × 6 = 2152.23 colonies.

Formula according to PN 89/Z-04008/08: CFU/m^3^ air = 82 × 10,000/63.5 × 30 × 0.2 = 2152.23 colonies.

**Figure 5:**
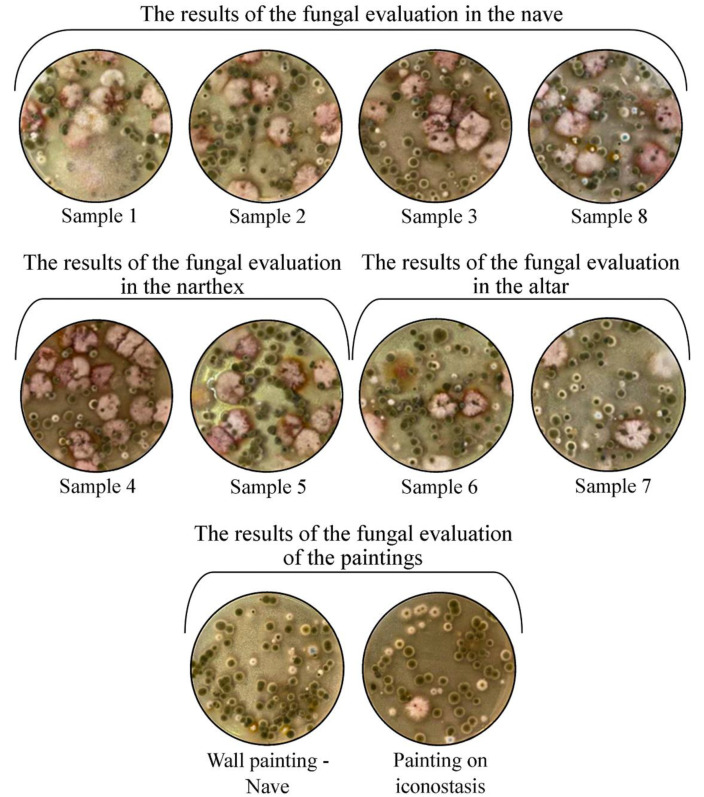
Appearance of fungal colonies at 72 h after sampling from the three rooms of the monument and from the inside paintings.

Usually, the dishes are monitored for a period of seven days for a complete analysis on the growth of fungal colonies. In this case, the mould colonies became macroscopically visible within 24 h, and on the fourth day the dishes were completely invaded. Thus, 82 different fungal colonies in the air were identified. These results correspond to the degree of contamination E, according to Table 1, respectively a very high degree of airborne fungal contamination, with over 2000 CFU/m^3^ air (Figure 5).

**Figure 6:**
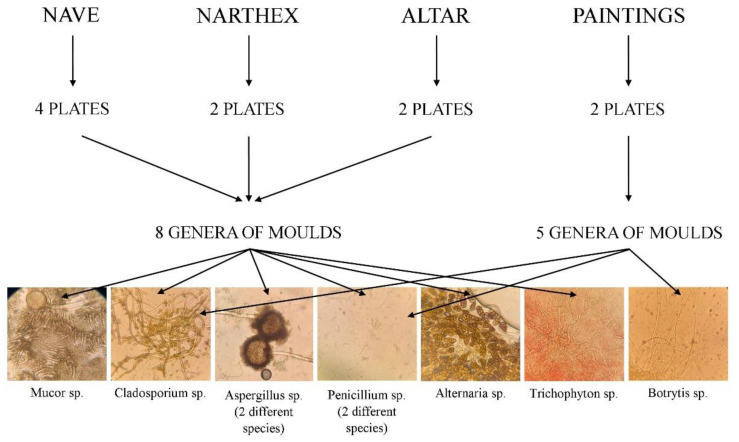
The genera of fungi identified inside the wooden church historical monument.

Macroscopic and microscopic examination of fungal colonies provided the necessary information on mold genera. Based on the macroscopic appearance of the colony (size, contour, shape, color, consistency, tendency to invade the culture medium) and the microscopic appearance (hyphae, pseudo-hyphae, conidia, sporangiospores, conidiophores, methules, phialides), we could establish the mold genera grown on culture media. Eight genera of mold were identified in the samples taken from the air. It was revealed that the same genera of fungi grew on the surface of the plates to a similar extent, their invasion being considerable. In the case of the two samples taken from the paintings in the nave and the iconostasis, the laboratory analysis revealed five genera of mold. Figure 6 shows that the invasion of the plates moderately increased.

The textile material made of hemp which constitutes the old painting is very much damaged by fungi present in indoor air and on the surfaces. These can contribute to the biodeterioration of the fabric over time, especially due to cellulose decomposing. The most active bio-deteriogens are: *Chaetomium*, *Fusarium*, *Aspergillus*, *Myrothecium*, *Cladosporium*, *Alternaria*, *Stachybotrys*, *Penicillium*, *Trichoderma*, *Pseudomonas*, *Arthrobacter*, *Sarcina* and *Streptomyces* [18,60,61,62].

**Figure 7:**
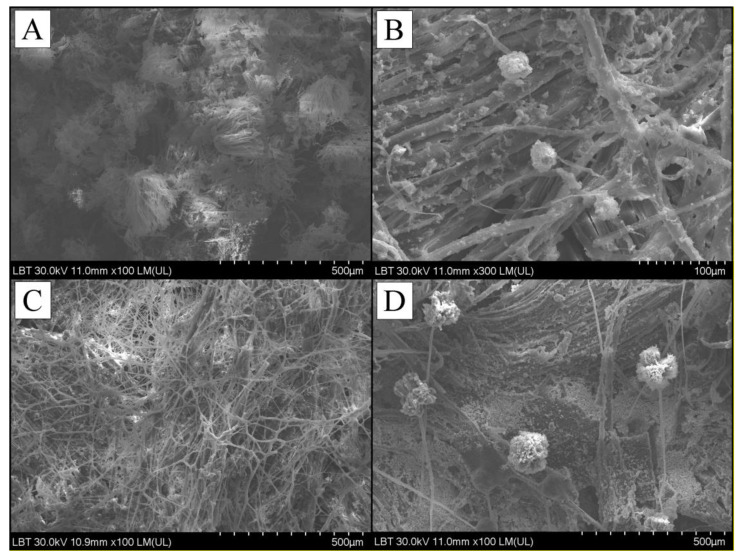
SEM analysis of sample number 1 of canvas from the walls of the wooden church. (**A**)—SEM image of the surface inoculated with fungi of *Aspergillus tamarii* species; (**B**)—SEM image of the surface inoculated with fungi of *Aspergillus tamarii* species after applying UVB treatment; (**C**)—SEM image of the surface inoculated with fungi of the species *Cladosporium cladosporiodes*; (**D**)—SEM image of the surface inoculated with fungi of the species *Cladosporium cladosporiodes* after application of UVB treatment.

**Figure 8:**
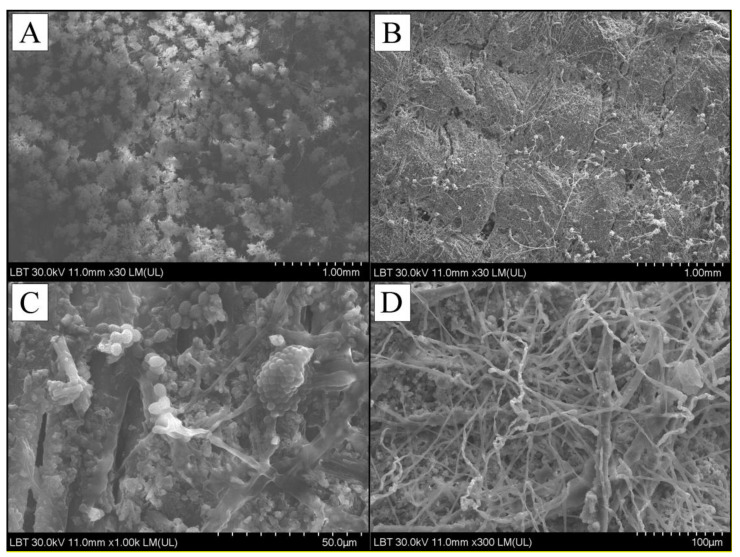
SEM analysis of sample number 2 of canvas from the walls of the wooden church. (**A**)—SEM image of the surface inoculated with fungi of *Aspergillus tamarii* species; (**B**)—SEM image of the surface inoculated with fungi of *Aspergillus tamarii* species after applying UVB treatment; (**C**)—SEM image of the surface inoculated with fungi of the species *Cladosporium cladosporiodes*; (**D**)—SEM image of the surface inoculated with fungi of the species *Cladosporium cladosporiodes* after application of UVB treatment.

Following the 30 min of UVB treatment a visible inhibition of fungal integrity was recorded. The SEM image shows a well developed fungal mat (panel A and C) and a clear degradation hife integrity and aggregation of fungal cells following treatment (panel C and D), on both samples of canvas, corresponding to Figure 7 and Figure 8. The results attested the fact that UVB irradiation can be a viable solution for cleaning fungal load textiles. However, in order to treat the objects belonging to the cultural heritage, which have a very high value, these treatments must not threaten the integrity of the materials. Therefore, the tests will continue in order to draw a strong conclusion regarding the effects that UVB irradiation can have on the long-term constitution of the material.

**Figure 9:**
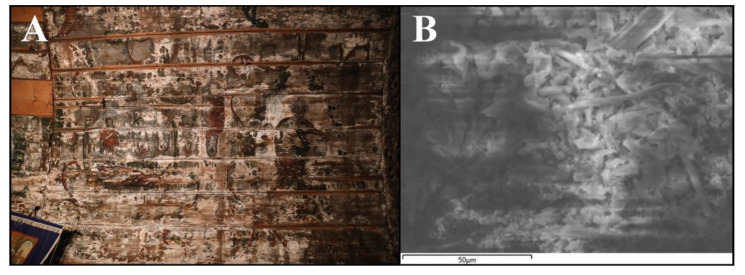
Details of the painting on canvas in the monument wooden church wall. *(***A**)—Painting on the hemp fabric on the walls of the monument wooden church; (**B**)—SEM image magnification ×50.

The analysis of the SEM images of the discussed samples shows interesting visual information regarding the fungal growth process on the natural hemp fibers from the wall paintings (Figure 9A,B) due to the presence of hyphae. Fungal growth can especially affect the surface of the fibers by premature dissolution [63]. Physical effects of biodegradation may be visible over time such as mass modification, aesthetic modification because of unwanted pigments or discoloration, structural damage, strength of fibers modifications, tensile, elasticity, abrasion resistance diminishing, fabric disintegration [64,65], as well as from a micromechanical point of view (rotting, breakdown, scratches, holes and cracking of the fibers).

## 5. Discussion

In addition to the destructive effects that fungi identified inside can have on the integrity of the monument itself and the objects inside, they also pose a danger to the health of visitors and those attending the religious services.

Thus, *Alternaria alternata*, *Chaetomium globosum*, *Aspergillus flavus* and *Penicillium oxalicum* fungal mechanisms in the deterioration of natural aged fibers are indicated as: “hydrolysis, oxidation, depolymerization and recrystallization” [66]. In order to determine the potential susceptibility to microbial degradation, respectively the bio-deteriorative potential of *Penicillium* spp. and *Arthrobacter* spp., on canvas paintings, the examination using FTIR-spectra (micro-Fourier Transform Infrared spectroscopy) revealed that only the inoculation of both species of indicated fungus can generate observable fibers’ biodeterioration effects [67].

*Alternaria* spp. may cause opportunistic human infections at skin level, subcutaneous infections, upper respiratory tract infections and onychomycosis. As Pastor and Guarro [68] point out in this regard, “immunosuppression is commonly associated with skin and subcutaneous infections as well as rhinosinusitis. The most significant risk factors for skin and subcutaneous infections are solid organ transplant and Cushing’s syndrome”. In immunocompetent subjects, *Alternaria* spp. does not represent a cause of infection as it is associated with allergic respiratory symptoms [68,69].

Previous investigations pinpointed in fungal species that belong to *Penicillium*, *Aspergillus*, *Cladosporium* and *Chaetomium globosum,* growth was limited to the surface of the natural fibers of the textile objects. In addition, the structural and physical changes induced to the fibers (e.g., discolouration and changes in surface, loss of strength and elongation) as well as in oxidation state-breakdown of molecular structure, are mainly shown on cellulotic fibres [18,70,71,72,73]. Tensile specific tests: Scanning Electron Microscopy, Raman spectroscopy, and Viscometric methods emphasized the fungal hyphal growth visualization on the natural fibers, the “fracture planes and splitting modes, etc., according to length of the fibers, conditions and time of exposure or other detected modifications in fibers’ morphology and the supermolecular structure” [74].

*Penicillium* spp. can cause infections and the principal ways of penetration into the human body are the respiratory system by inhalation. The characteristic pathologies are upper and lower respiratory tract infections; penetration into the human body can also be by ingestion orally and can cause various pathologies: keratitis, endophthalmitis, otomycosis, necrotizing esophagitis, endocarditis, peritonitis and urinary tract infections. Fungi belonging to the species *Penicillium marneffei* are pathogenic and specifically infect patients with Acquired Immunodefucuency Syndrome (AIDS), hematological and immunosuppressive malignancies [75,76].

*Aspergillus* may cause pathologies that specifically affect the respiratory system. The portal entrance into the human body is through inhalation. The infection can spread into the blood and reach skin, lungs, heart, brain, kidneys and affects people whose immunity is weakened. General signs and symptoms include: fever, chills, hemoptysis, inspiratory dyspnea, chest pain, and weight loss. In subjects with asthma, fungi can trigger a severe allergic bronchopulmonary aspergillosis, which include exacerbation of asthma attacks, fever and cough with sputum [77,78].

*Cladosporium* spp. is usually not pathogenic to humans. In prolonged exposure, an allergic reaction may develop in subjects with a history of atopy. Frequent symptoms are: dry and chapped skin, skin rash; stuffy nose, itchiness, nasal mucosa; tickly cough, dry cough, itchiness, laryngeal mucosa; eye secretions and conjunctivitis. In patients with known asthma, exposure to *Cladosporium* may exacerbate asthma attacks, with symptoms of increased wheezing, severe dyspnea associated with anxiety, compulsive cough and sleep disorders. Allergic fungal sinusitis can occur in subjects with prolonged exposure to *Cladosporium*, with the following specific symptoms: pain in the affected sinuses, nasal congestion, nasal secretions, diminished sense of taste or smell [79,80,81].

*Botrytis* spp. is slightly less pathogenic in humans and if inhaled can enter the body triggering, in the case of prolonged exposure, characteristic pathologies in the respiratory system, asthma attacks and the development of hypersensitivity pneumonitis. The clinical picture of the infection includes altered general condition and limited ability to mobilize the chest, dyspnea, dry cough and wheezing [82,83,84].

*Mucor* spp. can cause specific pathologies in humans. One of the gateways to the human body is again inhalation, which causes and develops infections in the lungs, sinuses and eyes. At the pulmonary level, patients develop a cough, dyspnea and fever. Symptoms of sinus mucor-mycosis include local pain, fever and headache. At the level of the skin, the pathway is via various traumas of the skin, such as cuts and scratches. Skin infections occur with changes in the color of the affected tissue, such as blisters [85,86].

In the case of Trichophyton, a fungus that was identified only on the paintings, the main mechanism of biodeterioration consists in keratinolysis [82]. This dermatophyte can cause infections in humans, which are transmitted through direct or indirect contact with an infected person or through conidia. It affects superficial areas of the body such as the epidermis (skin and skin appendages, hair, nails). The following mycoses are known: tines pedis (athlete’s foot), tinea corporis (body), tinea cruris (groin area), tinea capitis (scalp, hair), tinea barbae (beard) and tinea unguium (nails). Among the symptoms of mycoses are itching or burning sensation in the affected area, flaky or cracked skin, the onset of skin blisters, thickening and changing the structure and color of the affected nails, until the nail separates from its bed [87,88].

Most generated pathologies by the fungi are respiratory tract pathologies, which have an important socio-economic impact both due to their frequency and due to the influence they have on the quality of life of patients. In particular, people with frequent exposure to such an environment should follow a respiratory rehabilitation program that aims to reduce symptoms, optimize exercise capacity and reduce financial costs by stabilizing or limiting systemic manifestations of disease [89,90,91,92,93,94].

In terms of fungal removal, in addition to UVB treatments, essential plant extracts have proven to be very effective, as shown by Palla et al. [95] and Díaz-Alonso et al. [96] in their studies. The great advantage of these treatments is that they are non-invasive for the supporting materials, which is very important when it comes to old objects belonging to the cultural heritage. At the same time, it is essential to improve the air quality by means of instalation of heating, ventilation and air conditioning systems (HVAC). These technologies could improve the air change rate by bringing an additional supply of fresh air, besides that provided through the door, windows and cracks in the walls. Determining the number of air changes per hour (ACH) is very important when choosing an air purification system. According to the international standards in force [97], the number of air changes per hour (ACH) required in the case of churches should be between 8 and 12. The church in Boianu Mare, given that it has an interior volume of air of approximately 295 m^3^, would need HVAC technologies that generate a volumetric flow of at least 36.8 m^3^ /min to meet this standard. Installing mechanical ventilation will improve air quality, help prevent damage to structures and equipment, and improve the comfort of people in the church.

The conditions of the internal environment in a church must not only aim at creating comfort for users but also at adapting the optimal environment to the specifics of the elements in the location, which is often of a historical nature. The combination of cleaning techniques, air purification and natural ventilation often results in an excellent response. All these can ensure the comfort of visitors and compliance with international standards regarding the quality of the internal microclimate in heritage buildings.

## 6. Conclusions

According to the data obtained, we can state that the air inside the church (even if it is not used, being in a state of conservation) has a high degree of fungal contamination and is a risk factor for both human health and biodeterioration of valuable interior artwork. With respect to the examined surfaces, they also show a high degree of contamination, in accordance with the airborne microbial and fungal load. Five different genera of fungi were identified on the surfaces (two species of *Penicillium* spp., *Aspergillus* spp., *Cladosporium* spp., *Botrytis* spp.) and eight genera were identified in the air (two species of *Aspergillus* spp., two species of *Penicillium* spp., *Trichopyton* spp., *Mucor* spp., *Cladosporium* spp., *Alternaria* spp.). The development of these fungal colonies is favoured by temperature and RH, which are closely related to outdoor conditions, given the lack of heating, ventilation and air conditioning systems (HVAC). Temperature and RH were maintained throughout the monitoring period outside the accepted range and away from ideal values.

The effect of UVB was tested on a triplicate of plates for each fungal strain. A change in macroscopic morphology as compared to control was observed on all treated plates. SEM imaging revealed severe loss of cellular integrity following treatment. Thus, it can be concluded that the application of UVB in the doses indicated in this study has a clear antifungal effect on the *Cladosporium* sp. and *Aspergillus* sp. fungal genera, and especially on the species *Cladosporium cladosporiodes* and *Aspergillus tamarii*, very common in old heritage buildings. The results can be extrapolated to other genera of fungi, as well as to various objects belonging to the cultural heritage, assuming that the antifungal effects of UVB are to some extent similar.

The effects of the presented factors contribute to the creation of an unsuitable environment for parishioners and potential visitors. Over time this could lead to the deterioration of the structure and the materials of which the monument is made. To remedy the existing situation, it is necessary to identify non-invasive solutions for removing fungi and adjusting the values of the main indicators of the indoor microclimate. In this regards, the monitoring actions will continue in the wooden church, to obtain a complete overview of the changes in terms of microclimatic conditions and fungal load throughout the year. These variations will be analyzed in relation to the human health of those who are repeatedly exposed to this impure environment.

## Figures and Tables

**Table 1:** Assessment of the degree of air contamination in a room according to the health rules applied to non-industrial institutions (Source: Commission of European Communities [56]).

Degree of Air Contamination	Koch Sedimentation Method (CFU/m^3^ Air)
A—very low	<25
B—low	25–100
C—average	100–500
D—high	500–2000
E—very high	>2000

## Data Availability

The data presented in this study may be obtained on request from the corresponding author.
